# Osteoporosis of the vertebra and osteochondral remodeling of the endplate causes intervertebral disc degeneration in ovariectomized mice

**DOI:** 10.1186/s13075-018-1701-1

**Published:** 2018-09-10

**Authors:** Zhi-feng Xiao, Jian-bo He, Guo-yi Su, Mei-hui Chen, Yu Hou, Shu-dong Chen, Ding-kun Lin

**Affiliations:** 1 The Department of Spinal Surgery, The Second Affiliated Hospital of Guangzhou University of Chinese Medicine, No. 111, Dade Road, Yuexiu District, Guangzhou, 510120 China; 2 The Laboratory Affiliated to Orthopaedics and Traumatology of Chinese Medicine of Linnan Medical Research Center of Guangzhou University of Chinese Medicine, No. 12, Jichang Road, Baiyun District, Guangzhou, 510405 China; 3 Guangzhou University of Chinese Medicine, No. 12, Jichang Road, Baiyun District, Guangzhou, 510405 China

**Keywords:** Intervertebral disc degeneration, Osteoporosis, Ovariectomy, Endplate, Osteochondral remodeling, Microcomputed tomography

## Abstract

**Background:**

Studies on the relationship between osteoporosis and intervertebral disc degeneration (IVDD) are inconsistent. Therefore, we assessed whether IVDD is affected by vertebral osteoporosis in ovariectomized mice and investigated the underlying pathogenesis of IVDD related to osteoporosis.

**Methods:**

Thirty healthy female C57BL/6 J mice aged 8 weeks were randomly divided into two groups: a control group (sham operation, *n* = 15) and an ovariectomy group (OVX; bilateral ovariectomy, *n* = 15). At 12 weeks after surgery, the bone quantity and microstructure in the lumbar vertebra and endplate as well as the volume of the L4/5 disc space were evaluated by microcomputed tomography (micro-CT). The occurrence and characteristic alterations of IVDD were identified via histopathological staining. The osteoclasts were detected using tartrate-resistant acid phosphatase (TRAP) staining. Type II collagen (Col II), osterix (OSX), osteopontin (OPN), and vascular endothelial growth factor (VEGF) expression in the intervertebral disc were detected by immunohistochemical analysis.

**Results:**

OVX significantly increased the body weight and decreased the uterus weight. Micro-CT analysis showed that osteoporosis of the vertebra and osteochondral remodeling of the endplate were accompanied by an increase in the endplate porosity and a decrease in the disc volume in the OVX group. Likewise, histological evaluation revealed that IVDD occurred at 12 weeks after ovariectomy, with features of endochondral ossification of the endplate, loose and broken annulus fibrosus, and degeneration of nucleus pulposus. TRAP staining showed that numerous active osteoclasts appeared in the subchondral bone and cartilaginous endplate of OVX mice, whereas osteoclasts were rarely detected in control mice. Immunohistochemical analysis demonstrated that the expression of osterix was significantly increased, notably in the endplate of OVX mice. In addition, Col II was decreased in the ossification endplate and the degenerative annulus fibrosus, where OPN and VEGF expressions were elevated in OVX mice.

**Conclusions:**

OVX induced vertebral osteoporosis and osteochondral remodeling of the cartilaginous endplate contributing to the angiogenesis and an increase in porosity of the bone-cartilage surface, and also affected the matrix metabolism which consequently had detrimental effects on the intervertebral disc. Our study suggests that preserving the structural integrity and the function of the adjacent structures, including the vertebrae and endplates, may protect the disc against degeneration.

**Electronic supplementary material:**

The online version of this article (10.1186/s13075-018-1701-1) contains supplementary material, which is available to authorized users.

## Background

Lower back pain and spinal compression nerve pain are the major symptoms caused by intervertebral disc degeneration (IVDD) in the clinic [1, 2]. Spinal instability or disability is common in serious IVDD which leads to enormous human suffering and significant socioeconomic losses [3]. Unfortunately, there are no currently effective methods to repair IVDD [4] and disc resection with interbody fusion are often the final choice. Therefore there is an urgent need to explore the key mechanism of IVDD and to develop drugs for its treatment.

It has long been recognized that musculoskeletal degeneration disorders such as osteoporosis, IVDD, and osteoarthritis are a difficult focus in locomotor disease research. An intimate relationship between cartilage and subchondral bone has been proven in recent years. Anatomically, the vertebrae and the intervertebral discs are combined in bundles to form the motion segments of the spine. From the mechanical and biological points of view they are closely linked and are considered as a functional unit [5–7]. Although it is not fully clear whether it precedes or follows nucleus pulposus degeneration, the modic change in the endplate is an important feature of IVDD physiopathology [8]. Therefore, the health of the bone and its attached nonosseous tissues such as cartilage and disc are tightly associated. Research has shown that crosstalk between bone and cartilage is elevated in osteoarthritis where the coupling of bone and cartilage turnover is even aggravated [9–11]. Moreover, remodeling of the subchondral bone microstructure due to osteoporosis could further exacerbate experimental osteoarthritis [12]. All of this suggests that the subchondral bone is an indispensable factor in the process of osteoarthritis. In terms of IVDD, however, there is a lack of research on the pathological mechanisms of subchondral bone in IVDD, and it is also unclear how osteoporosis affects the nonosseous tissues such as the intervertebral disc.

Because of the structural similarities between joint and intervertebral discs [13, 14], it is well known that the intervertebral disc is a nonvascular structure and the exchange of substances between the intervertebral disc and the vertebra depends on the cranial and caudal endplates [15–17]. The endplate contains marrow contact channels which provide nutrients for the intervertebral disc and discharge metabolic waste through diffusion and liquid flow under cyclic loading [4, 18, 19]. Hence, changes in the vertebra microenvironment resulting from rapid bone turnover during vertebral osteoporosis and obstruction of marrow contact channels induced by calcification of the cartilaginous endplate may accelerate IVDD.

Recent studies have provided increasing evidence that osteoporosis is associated with the evolution of IVDD. The osteoporosis of vertebrae in postmenopausal women was correlated with IVDD [20, 21], and sex hormones can affect the severity of IVDD [21–23]. Furthermore, in osteoprotegerin (OPG) knockout mice, ossification occurred in the cartilage endplate and resulted in IVDD [24]. IVDD often occurs with osteoporosis of the vertebrae, indicating that the development of osteoporosis and IVDD might be a coupling process which could explain why postmenopausal women have more lower back pain than men. Some studies have reported the prospect of delaying the course of disc degeneration through improving bone metabolism and vertebral osteoporosis. For example, it was found that alendronate could retard the progression of lumbar IVDD in ovariectomized rats by improving the bone quality [25, 26]. Calcitonin could also suppress intervertebral disk degeneration and preserve lumbar vertebral bone mineral density and bone strength [27]. However, it is still puzzling that some clinical and epidemiological studies have shown that osteoporosis is inversely related to spinal degenerative diseases and IVDD [19, 28, 29]. These studies support osteoporosis in increasing endplate permeability and delaying IVDD [19]. In addition, radiographic features of lumbar disc degeneration were associated with an increased bone mineral density (BMD) in the spine [28, 30]. Thus, the relationship between osteoporosis and IVDD is still controversial and confusing. Consequently, whether a positive correlation exists between osteoporosis and IVDD and how the vertebral body affects the intervertebral disc are still undefined and remain to be further clarified.

Mice are commonly used as an animal model for osteoporosis and intervertebral disc degeneration [31–33]. Many studies have demonstrated that the ovariectomized mouse is a good model for postmenopausal osteoporosis [34]. Thus, the purpose of this study was to determine the roles of postmenopausal osteoporosis in IVDD and to further clarify its underlying mechanism by assessing the detailed pathological changes in L4–L5 spine motion segments including vertebrae, the endplate, and the intervertebral disc in ovariectomized mice.

## Methods

### Animals and designs

Female C57BL/6 J mice (8 weeks old) were purchased from the Animal Center of Guangzhou University of Chinese Medicine, Guangzhou, China (license number: SCXK (YUE) 2013–0034). The mice were housed in conditions of controlled temperature (22–25 °C) at 40–60% relative humidity with alternate day and night and were allowed food and water freely. The animals were randomly divided into two groups. The mice underwent either sham operation (control group, *n* = 15) or bilateral ovariectomy (OVX group, *n* = 15) while under anesthesia with 4% chloral hydrate at a dose of 400 mg/kg weight via intraperitoneal injection. The body weight of the mice was recorded weekly. The mice were sacrificed at 12 weeks after surgery, and L3–L6 spinal motion segments and hind limbs were harvested for subsequent experiments. All experimental protocols were approved by the Ethics Committee of Guangzhou University of Chinese Medicine and implemented according to the Guide for Use and Care of Animals.

### Microcomputed tomography analyses

L3–L6 spinal motion segments were harvested and immediately fixed in 4% neutral paraformaldehyde for 72 h at 4 °C. The samples were washed three times with phosphate-buffered saline (PBS) for 15 min each time. The L4–L5 segment of samples (*n* = 6) was measured by high resolution microcomputed tomography (micro-CT; Skyscan1172) according to a previously described protocol [31]. Briefly, the scanner was set at a voltage of 59 kV, a current of 100uA, and a resolution of 9 μm per pixel to measure the spinal segment. Images were reconstructed and analyzed using NRecon v1.6 and CTAn v1.9 software, respectively. Three-dimensional (3D) reconstruction images were obtained using CTvox v3.0. The coronal images of the L4/5 segment were used to perform 3D histomorphometric analyses of the intervertebral disc and cartilage endplate, while transverse images of L5 vertebrae were used to measure the vertebral body. The region of interest (ROI) of vertebrae, endplates, and intervertebral discs were depicted using CTAn v1.9. A total of 200 or 20 consecutive images of the ROI were respectively used to show the 3D reconstruction of the microarchitecture in the vertebra and endplate. The ROI of the intervertebral disc was shown as the mid-plane coronal images of the L4/5 segment. The disc volume was defined as the ROI covered by the entire invisible space between the L4/5 vertebrae. The cartilage endplate volume was defined as the visible bone plate volume that covers the vertebrae. Three dimensional structural parameters of vertebrae included the total volume of bone mineral density (BMDtv; reflecting bone mass per unit volume), percentage bone volume (bone volume (BV)/total volume (TV)), trabecular number (Tb.N; the inverse of the mean distance between the mid-axes of the structure), trabecular thickness (Tb.Th), trabecular separation (Tb.Sp; the average separation between the mid-axes), trabecular pattern factor (Tb.Pf; measuring the degree of convex surfaces and concave surfaces of the trabecular, where having many concave surfaces represents a well-connected spongy lattice, while more convex surfaces indicates a bad connectedness; the rising value represents the decrease in trabecular connectedness); connectivity density (CONN.D; reflecting the connection in the trabecular, with a lower value indicating increased interruption of the trabecular), and structural model index (SMI; reflecting the proportion of the plate and rod structure of the trabecular with a value range of 0–3 where a higher value indicates an increase in rod-shaped trabecular volume). The parameters of the endplate included the percentage bone volume (BV/TV), number of closed pores (Po.N(cl); representing the number of pores with a closed cavity in the endplate structure), open porosity (PO(op); open pore volume over total pore volume), and total volume of pore space (Po.V(tot)).

#### Histology and immunohistochemistry examinations

After fixation in 4% neutral paraformaldehyde for 72 h, the dorsal attachment including the vertebral arch/lamina and facet joint were removed from lumbar L4/5 spinal segments (*n* = 9) and were decalcified in 10% EDTA (pH 7.4) for 14 days at room temperature. The tibia received the same process. The samples were then dehydrated and embedded in paraffin. The L4/5 segments were sliced into 4-μm thick sections to perform hematoxylin and eosin (H&E) staining, safranin O and fast green staining, tartrate-resistant acid phosphatase (TRAP) staining, and immunohistochemistry.

H&E and safranin O staining were guided by the instructions of the reagent kits (Servicebio Biological Technology Co. Ltd., China). TRAP staining was handled according to the protocol of the staining kit (Solarbio Science & Technology Co. Ltd., China). Briefly, after dewaxing and hydration, slices were soaked in TRAP staining solution and incubated at 37 °C for 50 min. After washing in tap water, specimens were counterstained by methyl green.

Immunostaining was performed using standard protocols according to the Abcam/Santa Cruz official website (https://www.abcam.cn/protocols/ihc-tissue-processing-protocol and https://www.scbt.com/scbt/zh/resources/protocols/immunofluorescence-cell-staining). Briefly, sections were dewaxed and hydrated and then boiled in EDTA antigen repair solution (pH 8.0) for 20 min for antigen retrieval. Tissues were treated with 3% H_2_O_2_ to block endogenous peroxidase. The sections were then incubated with normal goat serum for blocking and subsequently with primary antibodies including mouse collagen II (Col II; Abcam, 1:200, ab34712), osterix (OSX; Santa Cruz, 1:200, sc-393,060), osteopontin (OPN; Santa Cruz, 1:200, sc-73,631), and vascular endothelial growth factor (VEGF; Santa Cruz, 1:200, sc-7269) at 4 °C overnight. Negative control slices were incubated in PBS without antibodies. For immunohistochemical staining, the rest of the procedures were manipulated according to the PV-6001 Two-Step IHC Detection Reagent instructions. After coloration with 3,3’-diaminobenzidine (DAB) solution (ZSGB-BIO Corporation, China), the sections were counterstained with hematoxylin and the yellow or brown color was considered as positive staining. For the immunofluorescent assay, the slides were incubated with the secondary antibody conjugated with fluorescence-Alexa Fluor® 555 (CST Corporation, USA, #4409) for 1 h avoiding light, followed by counterstaining with 4’, 6-diamidino-2-phenylindole (DAPI), and then detected under fluorescence microscopy (Olympus DP80, Japan). Positive staining in the disc was quantified using ImageJ Pro Plus software (Media Cybernetics, Baltimore, MD, USA).

### Statistics

All data were analyzed using the paired *t* test of SPSS 20.0 software (SPSS Inc., Chicago, IL). The comparisons of means were performed between the control and OVX groups. All data were checked for normality and homogeneity of variance and represent the mean ± standard deviation (SD). In all analyses, *p* < 0.05 was considered statistically significant.

## Results

### Macroscopic observation, body, and uterus weight of OVX mice

**Fig. 1:**
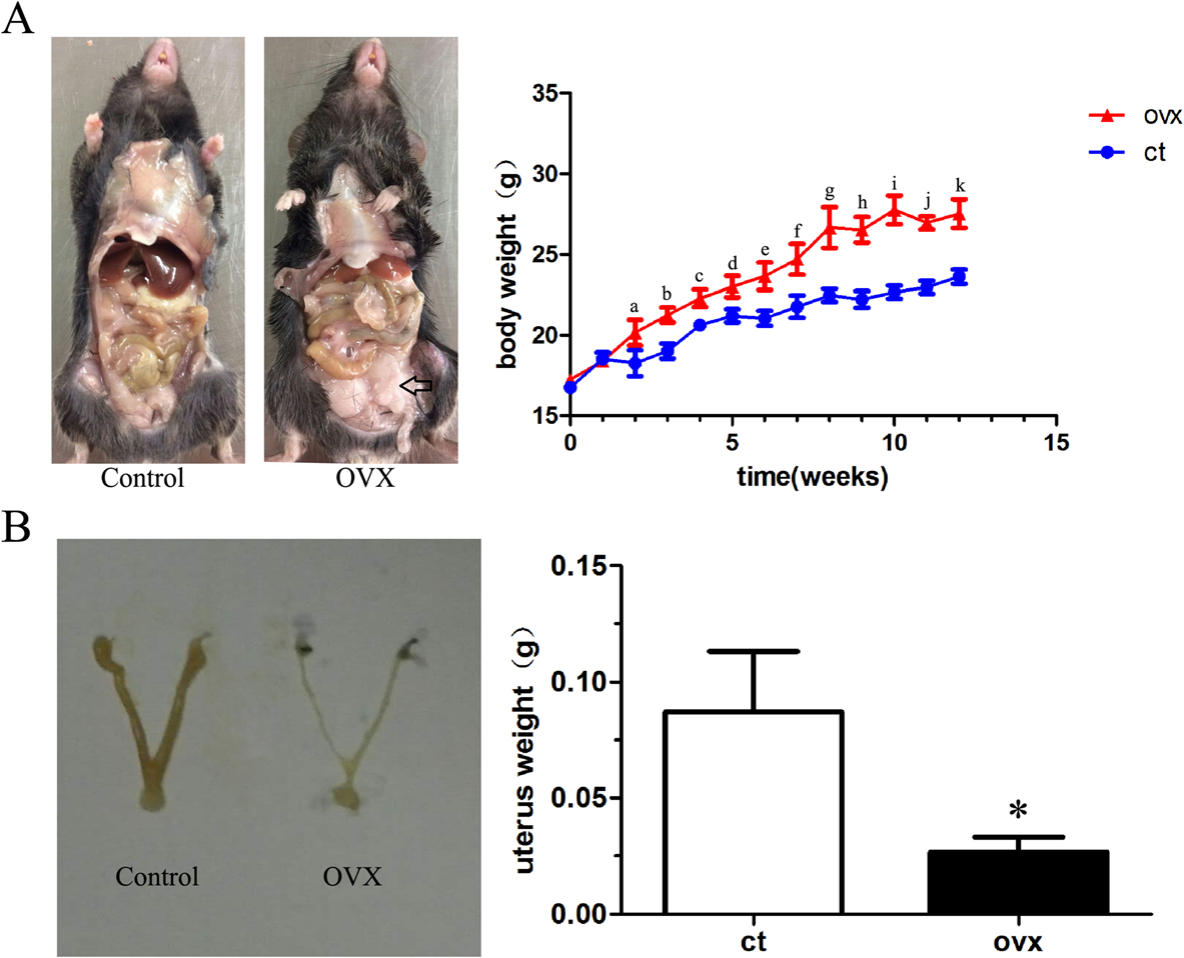
Gross macrographs and body and uterus weight changes of mice after ovariectomy. **a** There is a remarkable accumulation of fat in the abdominal cavity (black open arrow) and the body weight increase significantly over time in ovariectomized (OVX) mice. **b** The uterus is visibly atrophied in the OVX group and is significant lighter in weight compared with the control (ct) group. Data are shown as mean ± SD; *n* = 15 per group. ^a–k^*p* < 0.05 vs control group at 2–12 weeks for body weight; *p < 0.05 vs control group for uterus weight

The body weight of each animal was measured weekly in both the control and OVX groups. Results showed that the weight of the two groups increased over time, but was more significant in the OVX group from week 2 to week 12 (Fig. 1a; *p* < 0.05). At 12 weeks postsurgery, the abdominal cavity of OVX mice was filled with massive fat deposits and showed an obese body (Fig. 1a). In addition, a lack of estrogen from OVX resulted in atrophy of the uterus and significantly decreased uterus weight of the OVX group compared with the control group (Fig. 1b; *p* < 0.05).

### Changes in microarchitecture of the vertebra and endplate as well as increasing porosity lead to narrowing of the disc space in OVX mice

**Fig. 2:**
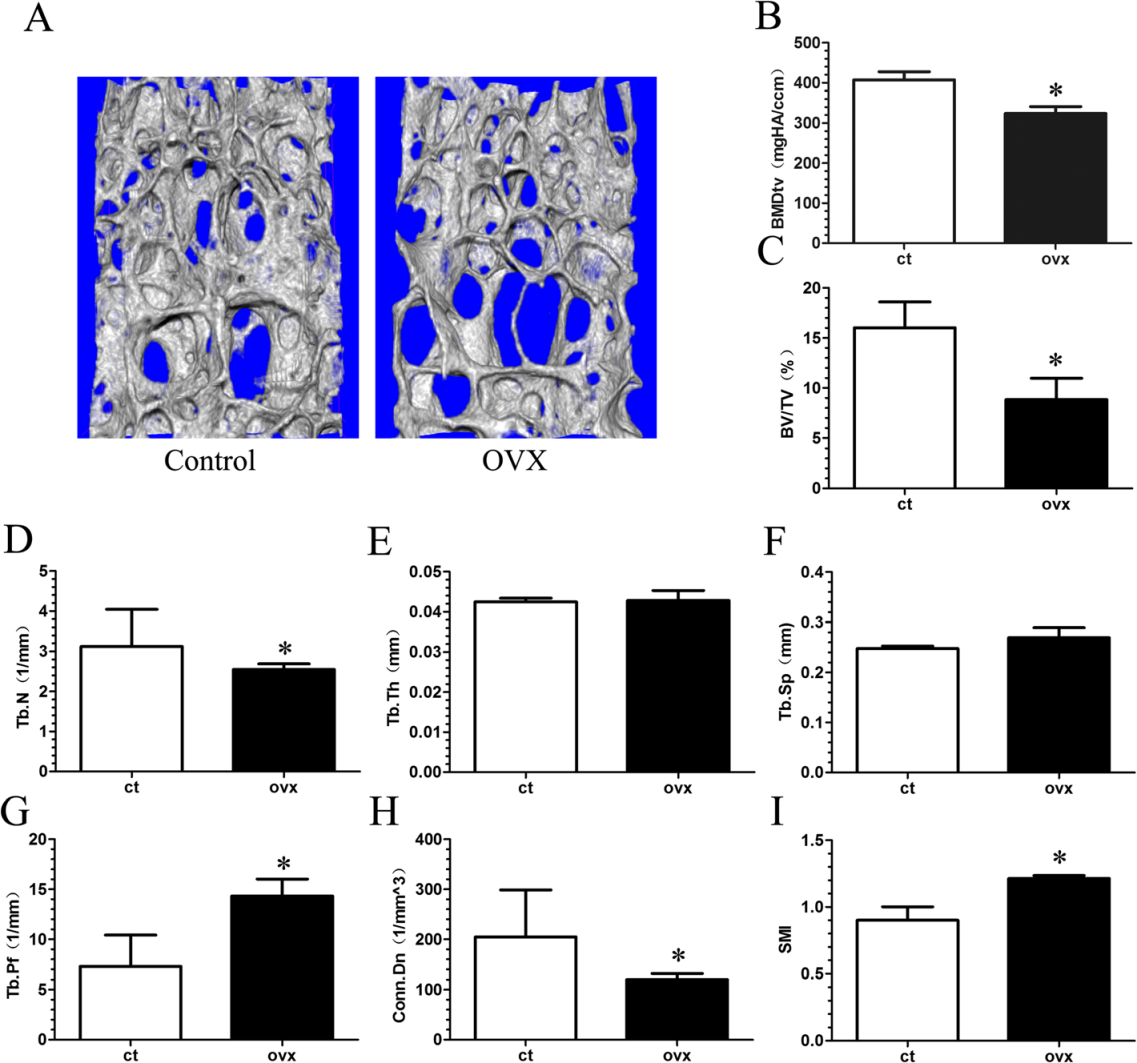
Changes in L5 total volume of bone mineral density (BMDtv) and microarchitecture parameters of the trabecular bone determined by micro-CT. **a** Representative three-dimensional images of trabecular bone. **b**–**i** The parameters of L5 trabecular bone. There is markedly decreased BMDtv, bone volume (BV)/total volume (TV), trabecular number (Tb.N; 1/mm), and connectivity density (CONN.D, 1/mm^3^), and increased trabecular thickness (Tb.Th; mm), and structural model index (SMI) in the ovariectomized (OVX) group when compared with the control (ct) group. Mice were analyzed at 12 weeks post-sham or OVX surgery, *n* = 6 per group. Data are shown as mean ± SD. *p < 0.05. Tb.Pf trabecular pattern factor, Tb.Sp trabecular separation

To determine if vertebral osteoporosis and endplate lesions, as well as disc changes, were presented in OVX mice, micro-CT analysis was performed. Sparse trabeculae were displayed in the OVX group (Fig. 2a and Additional file 1: Figure S1). Quantification of the trabecular structures revealed that BMDtv, BV/TV, Tb.N, and Conn.Dn in the OVX group were significantly decreased when compared with the control group (Fig. 2b–d, h; *p* < 0.05), while the Tb.pf and SMI of the OVX group were markedly higher than those of the control group (Fig. 2g, i; *p* < 0.05). Although no significant difference was observed for Tb.Th and Tb.sp. between the two groups, the OVX mice showed a slightly higher value for Tb.sp. (Fig. 2e, f).

**Fig. 3:**
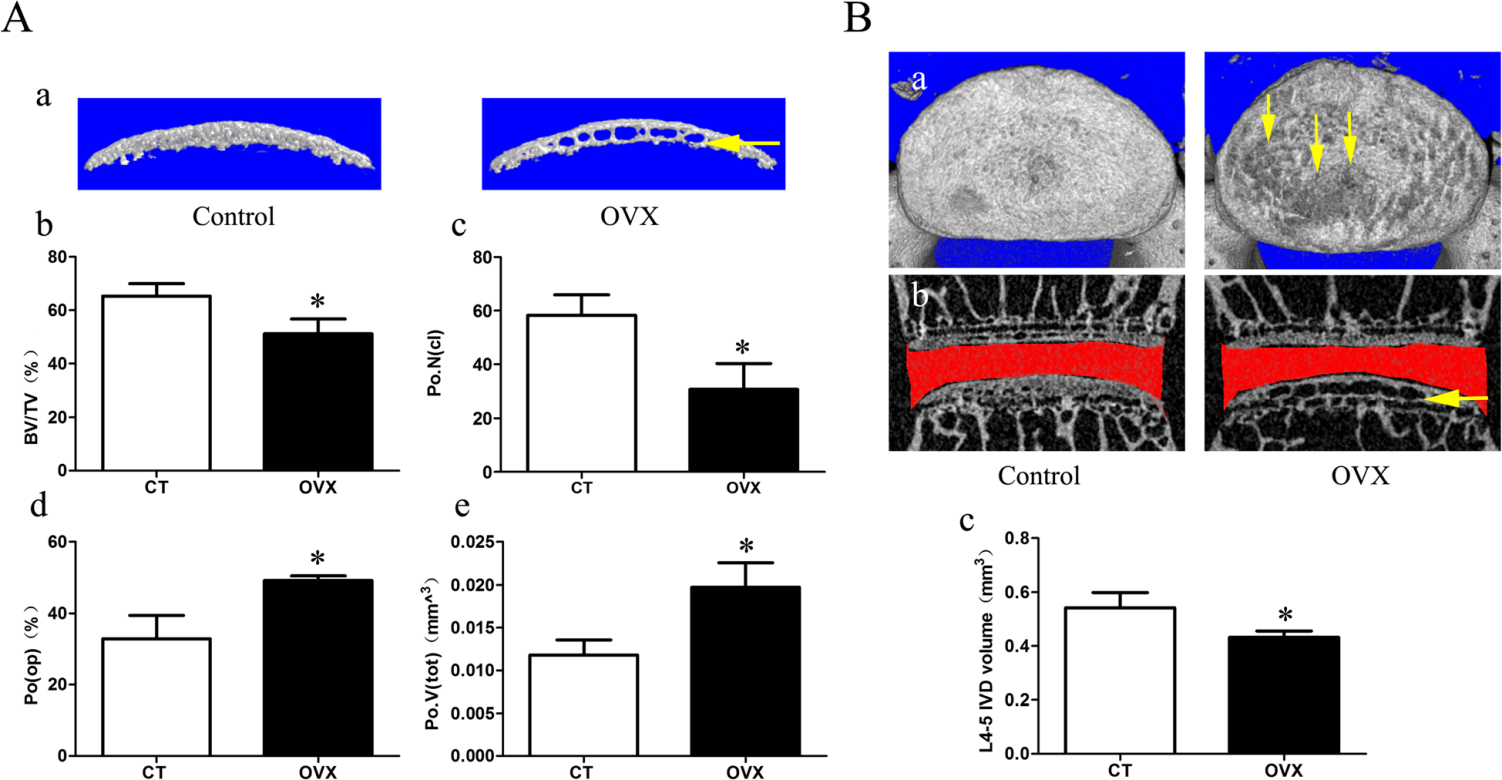
Changes in microarchitecture, porosity of L4/5 caudal endplate, and disc volume quantified by micro-CT analysis. **A a** Three-dimensional images and parameters of caudal endplate. Results showing that increased cavities in OVX mice (yellow arrow) indicate osteochondral remodeling of the endplate. **b–e** Markedly decreased bone volume (BV)/total volume (TV) and number of closed pores (Po.N(cl)) and increased open porosity (Po(op)) and total volume of pore space (Po.V(tot)) are shown in the ovariectomized (OVX) group. **B a** The top view of the caudal endplate showing a higher surface porosity in OVX mice (yellow arrows). **b,c** Quantification of disc volume by micro-CT showing a significant decrease in OVX mice. The ROI of the disc is indicated by the red color. Data are shown as mean ± SD, *n* = 6 per group. *p < 0.05. CT control, IVD intervertebral disc

Moreover, a large number of cavities was found in the endplate of the OVX group (Fig. 3A and Additional file 1: Figure S1B). To confirm the degree of porosity in the endplate, we examined the micro-CT parameters of the caudal endplate. The parameters showed that the BV/TV and Po.N(cl) were significantly decreased (*p* < 0.05), while the PO(op) and po.V(tot) were significantly increased (*p* < 0.05), suggesting an increasing endplate porosity in the OVX group. Additionally, from the top view of the caudal endplate, there were markedly higher pores on the surface of the endplate in the OVX mice and most of them were located in the central area corresponding to the nucleus pulposus (Fig. 3B-a). Furthermore, the intervertebral disc space of the OVX mice were narrowed (Fig. 3B-b and Additional file 1: Figure S1B). Meanwhile, the OVX group showed a significantly smaller intervertebral disc volume than the control group (Fig. 3B-c; *p* < 0.05).

### Osteoporosis with osteochondral remodeling of the endplate causes disc degeneration in OVX mice

**Fig. 4:**
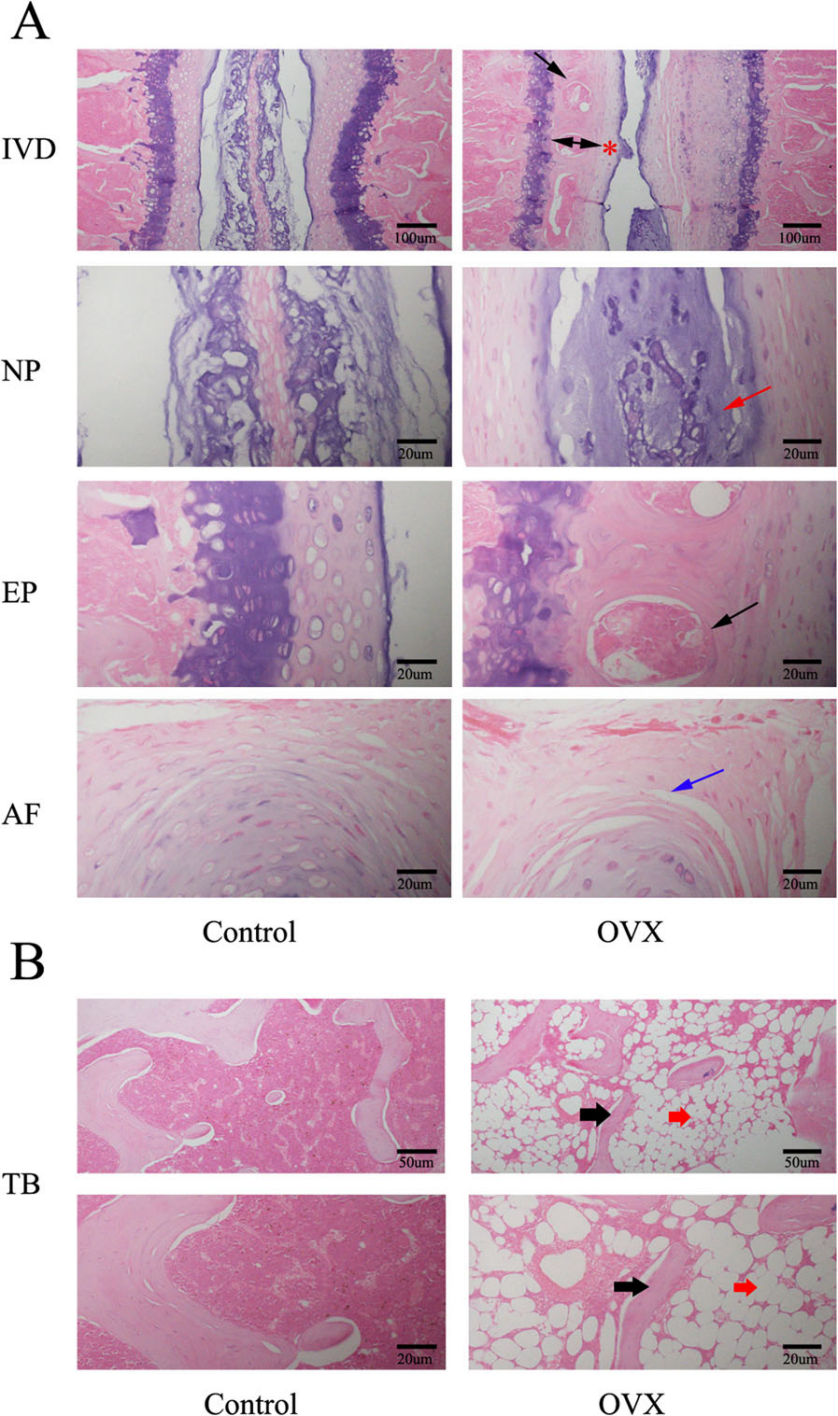
Representative images of H&E staining of the intervertebral disc (IVD) and tibia (TB). **a** Panoramic images of IVD pathology and higher magnification of the endplate (EP), nucleus pulposus (NP), and annulus fibrosus (AF). Ossific nodules (black arrows) in the endplate along with thickening of bony endplate (double arrow) and thinning of the cartilage endplate (red asterisk) were indicated in ovariectomized (OVX) mice. In addition, reduction of notochord cells, degeneration of nucleus pulposus (red arrow), and cleft formation within the annulus fibrosus (blue arrow) appeared in OVX mice. **b** Images of tibial pathology. Black arrows demonstrate slender trabecular bone and red arrows indicate fat droplets in the bone marrow of OVX mice. *n* = 9 per group; scale bars = 100 μm, 50 μm, and 20 μm as indicated

To test if the disc space stenosis in the OVX mice was related to osteoporosis and the accelerated osteochondral remodeling, we performed histological studies. H&E staining of tibia further confirmed osteoporosis in the OVX group, in which trabeculae were sparse and diminished (Fig. 4b). The bone marrow was almost replaced by adipose tissue (Fig. 4b).

**Fig. 5:**
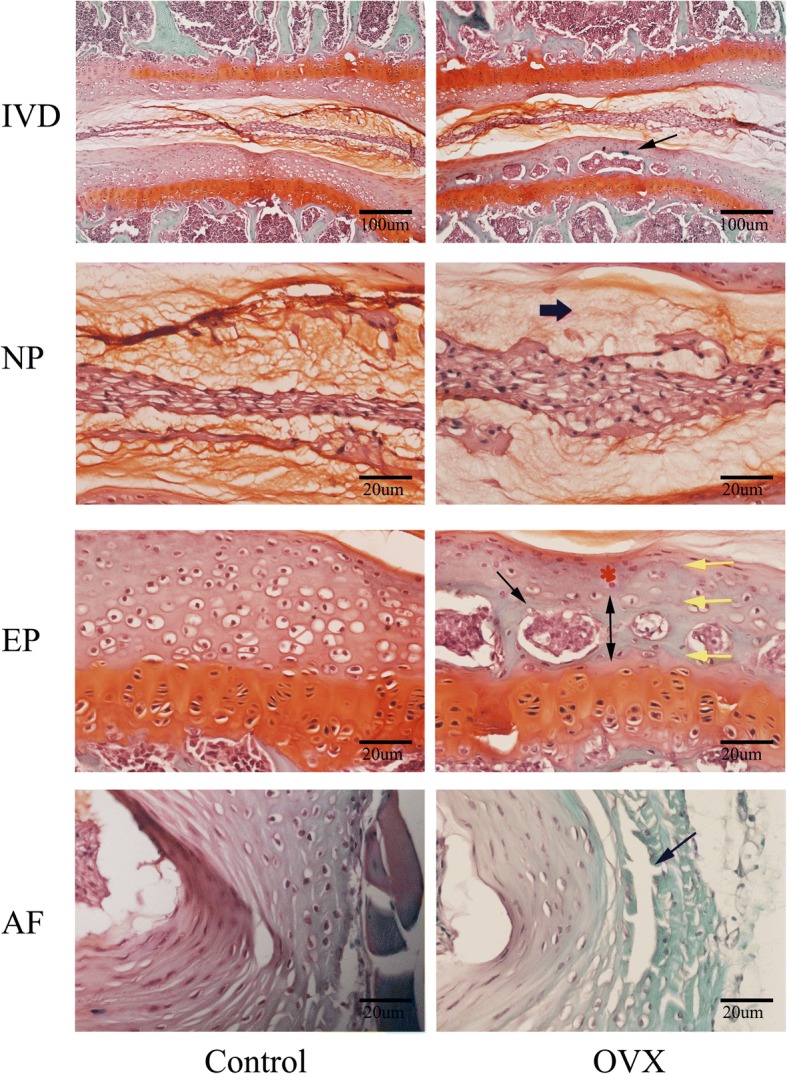
Representative images of safranin O and fast green staining of the intervertebral disc (IVD). This showed consistent results with H&E staining. Specially, thickening of the bony endplate (EP; double arrow) accompanied by accelerated osteochondral remodeling (black arrows) and duplication of tidemarks (yellow arrow) were displayed more clearly in the EP of ovariectomized (OVX) mice by safranin O fast green staining. Moreover, reduction of aggrecan in the nucleus pulposus (NP), as indicated by a paucity of safranin O staining (blue thick arrow), cleft/crack formation in the annulus fibrosus (AF) (blue arrow), and loss of cells indicate intervertebral disc degeneration in OVX mice. *n* = 9 per group; scale bars = 100 μm and 20 μm as indicated

Histological findings of the intervertebral disc volume showed that the nucleus pulposus contained abundant notochordal cells surrounded by large zones of extracellular matrix, and the cartilaginous endplates were hyaline cartilages composed of chondrocytes in the control group. In contrast, in the OVX group, the discs showed degenerative changes and reduction in the nucleus pulposus and were comprised of relatively few, clustered doublets of chondrocyte-like cells (Fig. 4a). Proteoglycan loss could be also found in the nucleus pulposus of OVX mice with pale safranin-O staining (Fig. 5). Moreover, ossification of the cartilaginous endplate occurred in the OVX group (Figs. 4a and 5). Safranin O and fast green staining suggested that endplates underwent endochondral ossification at 12 weeks postsurgery, indicated by the erosion of the wavy tidemark (Fig. 5) and green-stained bone matrix surrounding the cavities in OVX mice relative to the control group (Fig. 5). The endplate structure showed damage, further calcification and ossification, and eventually became a bony structure. Bony tissues contained bone marrow, hematopoietic lineage cells, and mineralized bone, and appeared more obvious in the deep zone of the middle cartilaginous endplate (Figs. 4a and 5). However, the superficial cartilage endplate became markedly thinner and had fewer active cells (Figs. 4a and 5). Furthermore, an increased number of clefts formed in the annulus fibrosus of OVX mice with collagen disarrangement and cell reduction, and even loose and broken lamella (Figs. 4a and 5).

#### Increasing bone turnover accompanied by osteochondral remodeling exists in OVX mice

**Fig. 6:**
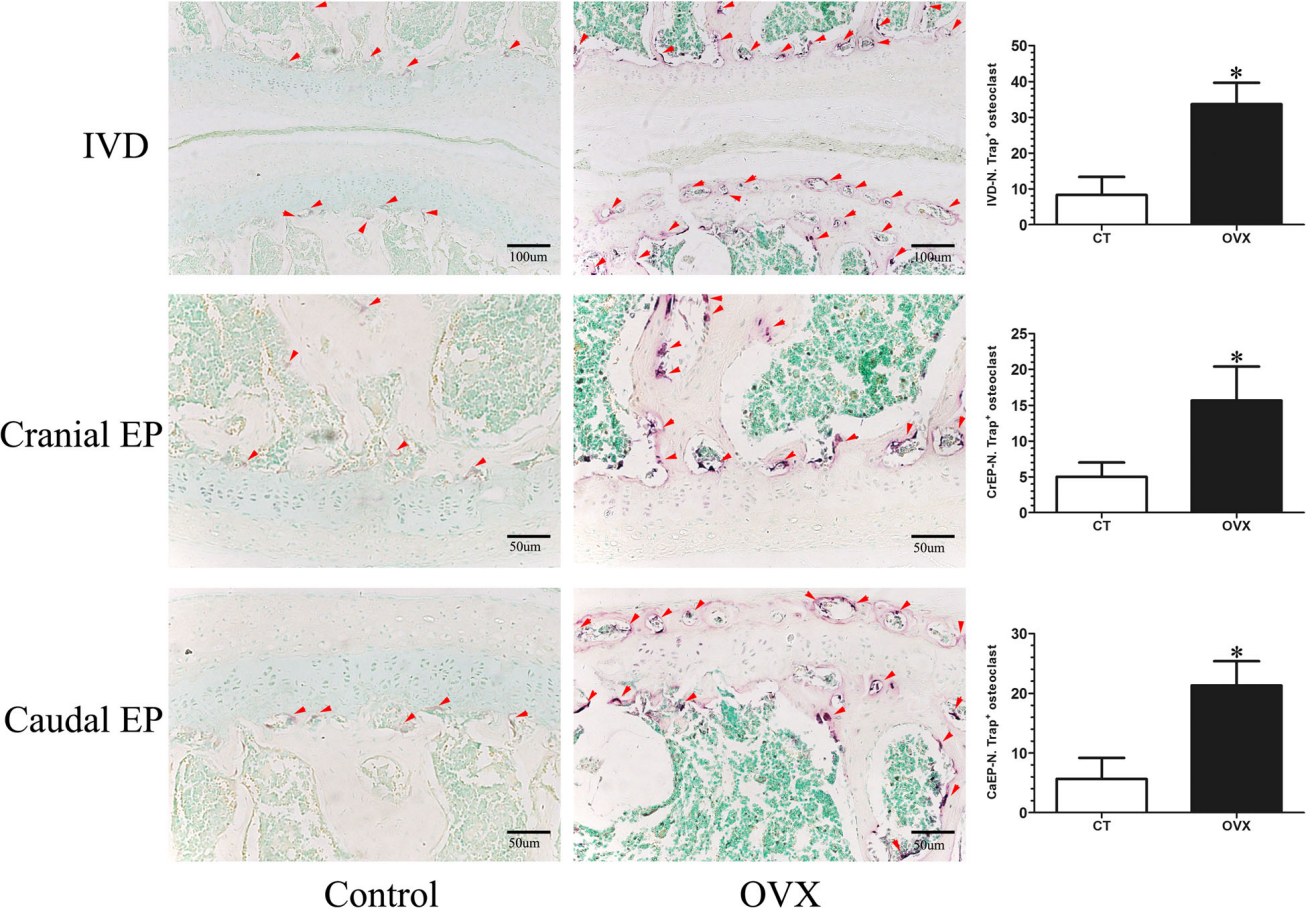
Tartrate acid phosphatase (TRAP) staining of L4/5 coronal sections. The osteoclasts were obtained by counting the number of TRAP-positive staining cells (N. Trap^+^). A few TRAP-positive cells (purple; red arrows) were distributed on the surface of the trabecular of the subchondral bone and were rarely detected in the endplate of control (CT) mice. However, the TRAP-positive cells (purple; red arrows) significantly increased in the subchondral bone and were obviously noted in the endplate of ovariectomized (OVX) mice, suggesting osteoclast activity increases after OVX. Data are shown as mean ± SD, *n* = 6 per group. *p < 0.05; scale bars = 100 μm and 50 μm as indicated. CaEP caudal endplate, CrEP cranial endplate, IVD intervertebral disc

**Fig. 7:**
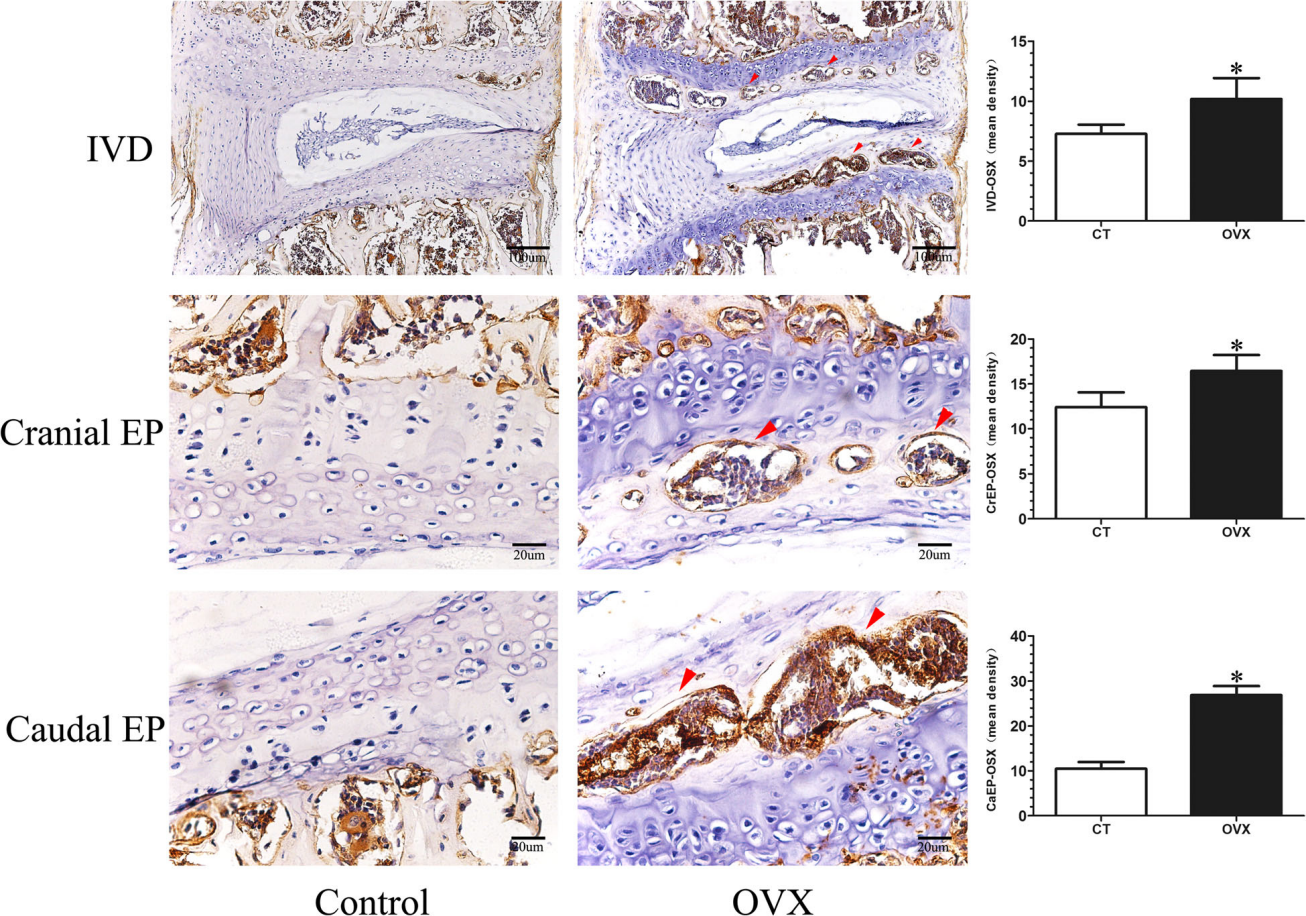
Representative sections of immunohistochemistry of osterix in the mid-sagittal plane. The mean integrated optical density (mean density) was obtained to assess the expression of osterix (OSX). Little osterix expression could be observed in the intervertebral disc (IVD) of control (CT) mice, whereas a significant increased osterix expression (red arrows) was found in the subchondral bone and endplate of ovariectomized (OVX) mice. Data are shown as mean ± SD, *n* = 6 per group. *p < 0.05; scale bars = 100 μm and 20 μm as indicated. CaEP caudal endplate, CrEP cranial endplate

TRAP and OSX were detected to reflect the bone turnover of the intervertebral disc volume. TRAP staining showed that many activated osteoclasts were widely located on the trabecular surface of subchondral bone and were significantly increased in the cartilaginous endplate of OVX mice (*p* < 0.05), but there were only a few osteoclasts in the control mice (Fig. 6). Simultaneously, OSX expression was significantly elevated in the OVX mice (*p* < 0.05), especially in the ossification region of the endplate which was consistent with the area of osteoclast activation (Fig. 7).

#### Decreasing Col II and increasing OPN and VEGF are expressed in the degenerative disc of OVX mice

**Fig. 8:**
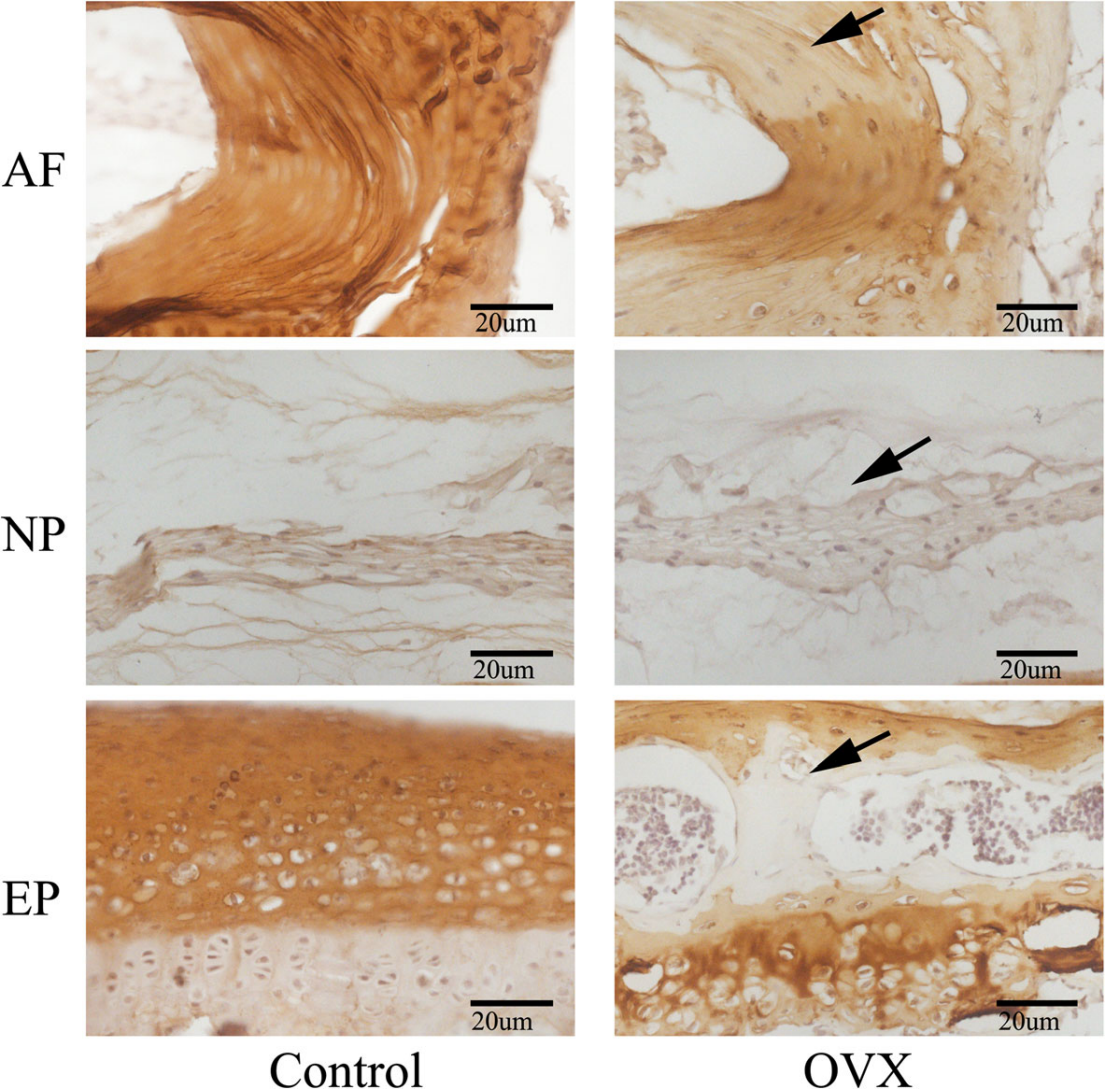
Representative sections of immunohistochemistry of Col II. Positive immunostaining was noted as brown staining. Black arrows indicate loss of Col II, especially in the ossification area of the cartilaginous endplate (EP) and the outer layer of the annulus fibrosus (AF) in ovariectomized (OVX) mice. *n* = 9 per group; scale bars = 20 μm as indicated. NP nucleus pulposus

**Fig. 9:**
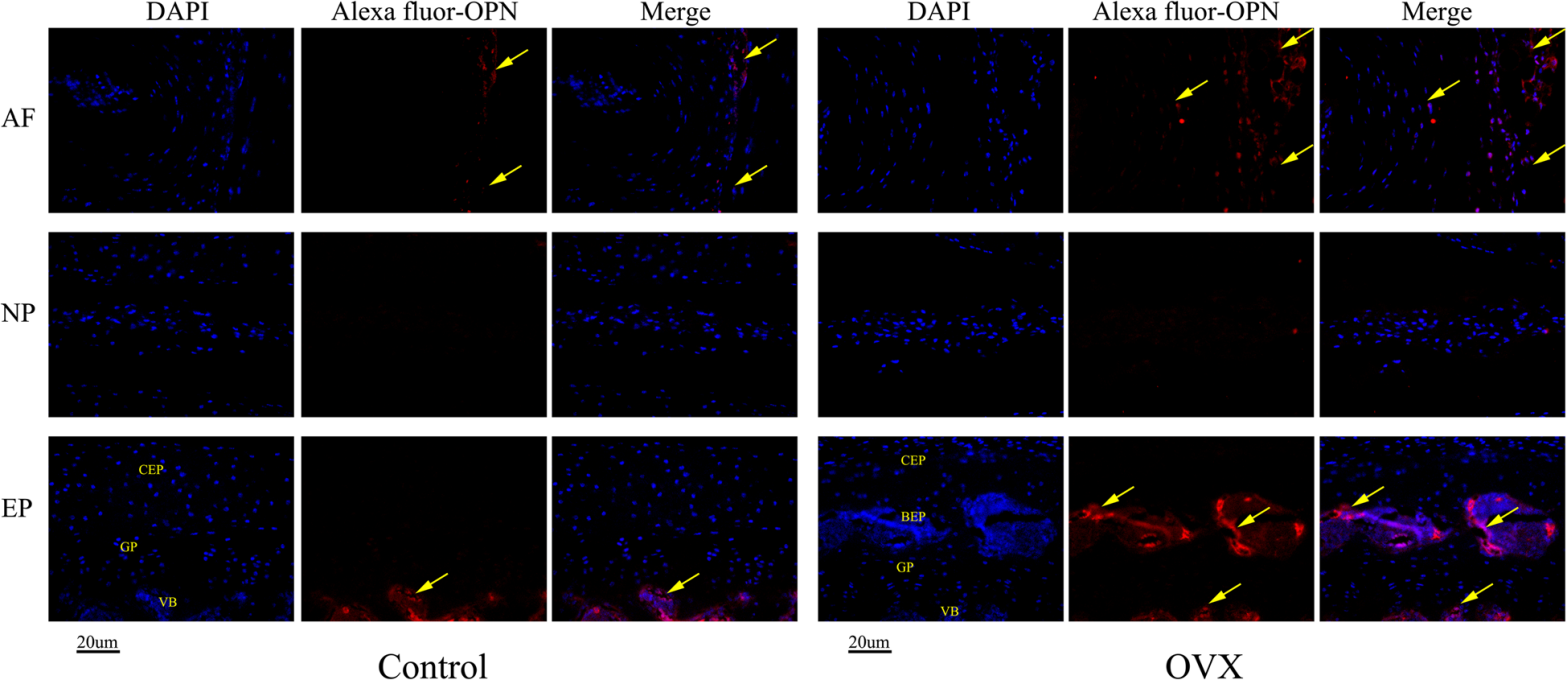
Immunohistochemical staining of osteopontin (OPN). DAPI stains nuclei blue and OPN expression was detected as red. Both control and ovariectomized (OVX) mice have OPN expression in the vertebral body (VB) and outer annulus fibrosus (AF), but it was almost undetectable in the nucleus. However, remarkable expression of OPN was found in the bony endplate (BEP) and outer AF in OVX mice. Meanwhile, the expression of OPN was also detected in the inner AF. *n* = 9 per group; scale bar = 20 μm as indicated. CEP cartilaginous endplate, EP endplate, GP growth plate, NP nucleus pulposus

**Fig. 10:**
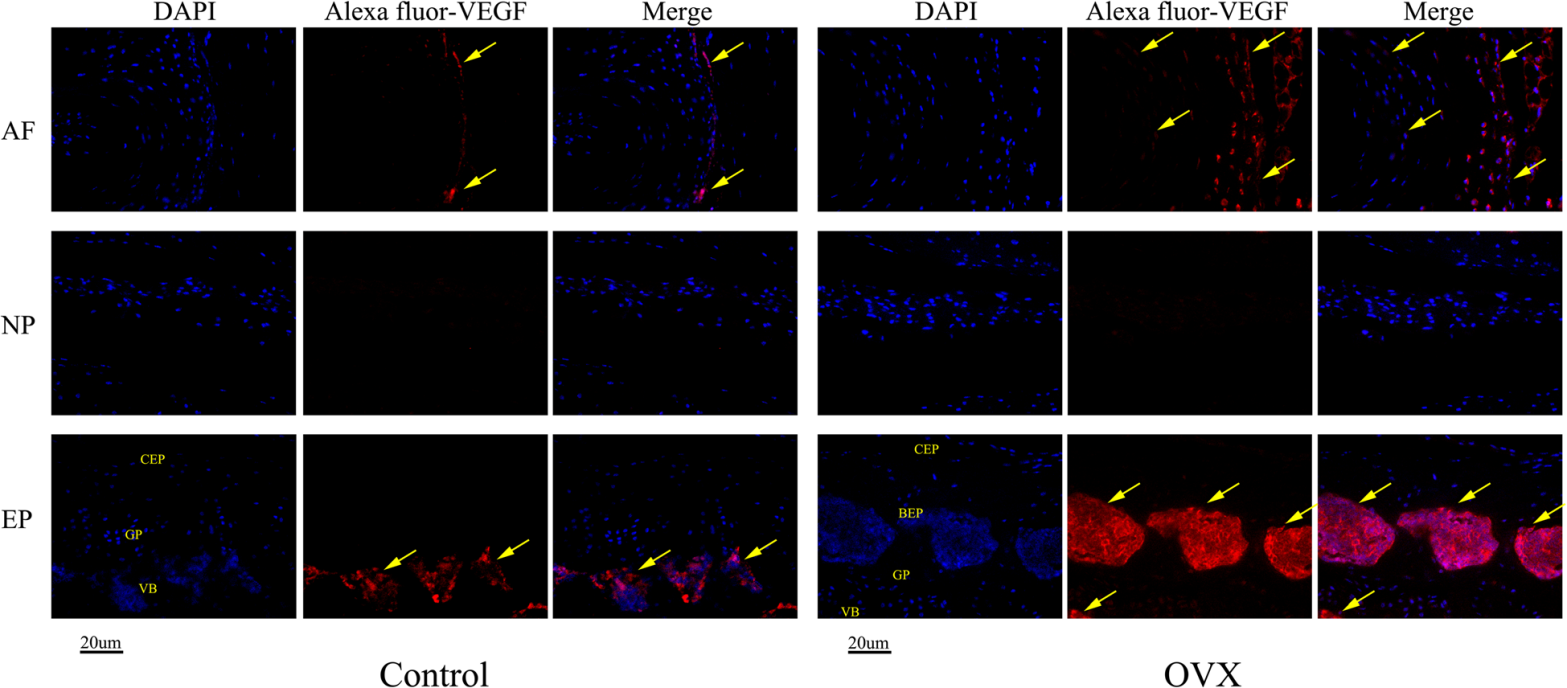
Immunohistochemical staining of vascular endothelial growth factor (VEGF). Consistent with OPN expression, a large amount of VEGF expression was observed in the outer layer of the annulus fibrosus (AF) and the bone marrow of the bony endplate (BEP) indicating that vasculogenesis and angiogenesis are associated with ossification of soft tissue and play an important role in the process of IVDD. *n* = 9 per group; scale bars = 20um as indicated. CEP cartilaginous endplate, EP endplate, GP growth plate, NP nucleus pulposus, VB vertebral body

Immunohistochemistry and immunofluorescence assays were performed to assess the protein levels of Col II, OPN, and VEGF in the intervertebral disc. As seen in Fig. 8, the changes in the organization of Col II were commonly detected in the cartilage. The expression of Col II is significantly decreased in the discs of OVX mice, especially in the endplate ossified zone and outer layer of the annulus fibrosus, and their spatial arrangement has changed in the remainder of the endplate (Fig. 8). However, OPN (an osteogenic marker protein) and VEGF (a cytokine associated with angiogenesis), which are closely associated with endochondral ossification and rarely expressed in normal discs, were markedly increased in the endplate and annulus fibrosus in OVX mice (Figs. 9 and 10). Interestingly, decreased Col II expression and increased OPN and VEGF expression was colocalized in the endplate ossified areas and inner annulus fibrosus, suggesting that osteochondral remodeling and abnormal angiogenesis in the avascular soft disc was a coupling process and may be an important link to disc degeneration in OVX mice.

## Discussion

IVDD and osteoporosis are the most common degenerative diseases in the spine, both of which are often accompanied with the other. However, the detailed relationship between them is not clear. In the present study, we evaluated the effects of estrogen deficiency on the bone mass and microarchitecture in vertebrae, the microarchitecture and porosity of the endplate, and the histopathology of the adjacent intervertebral disc to elucidate the possible relationship between osteoporosis and IVDD and to explore the superficial mechanism of IVDD associated with osteoporosis.

Our study showed that OVX could cause vertebrae osteopenia. Additionally, OVX promoted bone turnover and osteochondral remodeling at the junction of the vertebra and intervertebral disc, leading to an increased ossification and hypertrophy of the endplate, abnormal pores within the cartilaginous endplate, high porosity between the vertebra and intervertebral disc, and narrowing of the intervertebral disc space. Therefore, OVX exerted a detrimental effect on subchondral bone structure, particularly in the subchondral plate, which was closely related with disorders of the overlying cartilaginous endplate and played a crucial role in the development of IVDD.

At the same time, decreasing proteoglycans in the nucleus pulposus, increasing cracks within the annulus fibrosus, and osteochondral remodeling of the endplate could be also found in the intervertebral disc histomorphology of OVX mice. TRAP staining showed that the osteoclasts in the subchondral bone were significantly increased and particularly appeared in the endplate. The immunohistochemistry showed a corresponding increase in OSX expression, indicating that the ovariectomy induced a fast bone turnover which led to the structural remodeling of the endplate and changes in the porosity. It has been reported that there is a significant correlation between the effective permeability and marrow contact channel or porosity of the endplate [35, 36]. Thus, the increase in permeability induced by postmenopausal vertebral osteoporosis and endplate remodeling might be the most important factor contributing to the lesions of the soft tissue of the intervertebral disc in OVX mice. Additionally, the results of immunohistochemistry showed that OVX weakens the expressions of Col II and upregulates OPN and VEGF expressions in the endplate and annulus fibrosus, suggesting that abnormal ossification and angiogenesis are involved in the process of IVDD related to osteoporosis.

Estrogen as an endocrine hormone influences the metabolism of various tissues and organs in the body, such as the rich collagenous tissues of bone, cartilage, disc, artery, and skin, etc. [23]. It has been clearly shown that a rapid decline of estrogen levels is an important factor for osteoporosis in postmenopausal women. Insufficient estrogen caused by OVX is the common method to mimic postmenopausal status and can effectively induce osteoporosis. Many studies have confirmed that ovariectomy could cause osteoporosis in various animals, such as rats, mice, monkeys, and so forth [25, 32, 37]. In the current study, our results show that ovariotomy caused an increased body weight with abdominal fat accumulation but a decreased uterine weight with a severe atrophy in morphology. Furthermore, a mass of fat in the bone marrow of the tibia and thin trabeculae were observed in OVX mice, which supports that estrogen deficiency may induce adipogenic differentiation of bone marrow stem cells, but not osteogenesis. Moreover, the poorer results for BMDtv and the bone parameters including BV/TV, Tb.N, Tb.pf, CONN.D, and SMI were seen in the L5 vertebra of the OVX group when compared with the control group suggesting a deterioration in bone quality and quantity due to ovariectomy, consistent with previous studies [32, 37, 38]. Progressively, we investigated the effects of OVX on the intervertebral disc. As expected, OVX mice showed increases in the osteochondral remodeling and porosity of the endplate accompanied by decreases in the height and volume of the intervertebral disc, loss of proteoglycans and cells, and the formation of clefts, suggesting that ovariotomy effectively accelerates the deterioration of the endplate and induces IVDD. Considering that the intervertebral disc is avascular, material exchange with the vertebrae mainly relies on the endplate. Thus, it can be speculated that some effects of estrogen on the intervertebral disc are indirect. The initial occurrences of osteoporosis and cartilaginous endplate remodeling and subsequent disorders of intervertebral disc metabolism may be a reasonable explanation for the induction of IVDD by estrogen deficiency.

Many risk factors have been found to be involved in the IVDD process, including age, sex, injury, obesity, genetic predisposition, immune, nutrition, inflammation, and mechanical factors [7, 24, 39]. Increasing evidence indicates that IVDD is associated with the disruption of an intact spinal structure, such as adjacent structures including the vertebra and endplate [14, 16, 17, 22]. In recent years, the important role of subchondral bone in the development of osteoarthritis has been increasingly recognized [40]; in fact, it is the same with IVDD. Changes in vertebral strain energy were correlated with increasing Schmorl’s nodes in multilevel lumbar disk degeneration [41]. The changes in vertebral-endplate subchondral bone signal detected by magnetic resonance imaging (MRI) may serve as an ‘active discopathy’ judgment [8]. Therefore, as the bridge of communication between the vertebra and the intervertebral disc, the integrity of the endplate may be the key factor affecting the intervertebral disc [42, 43], and the deterioration of subchondral bone may be the trigger of IVDD. Interestingly, in some studies, ossification of the endplate with a reduction in porosity and permeability accelerated degeneration of the intervertebral disc during the osteoporosis process [17, 38]. It has been shown by a dynamic contrast-enhanced MRI study that ovariectomy induces a decrease in the second wash-in phase, indicating that the diffusion between the vertebra and the disc was impaired as a result of ovariectomy [44]. On the contrary, other studies have concluded that osteoporosis increases the porosity and permeability of the endplate, leading to a delay in disc degeneration [19]. Indeed, these conclusions are derived from the theory that nutrient acquisition of the intervertebral disc relies on endplate permeability. However, our study has found that endplate osteochondral remodeling causes a high endplate porosity/permeability leading to disc degeneration, and this may be associated with antigen exposure, immune inflammation, and loss of nucleus pulposus osmotic pressure in the intervertebral disc due to the increased endplate porosity. TRAP staining showed that a large number of activated osteoclasts appeared in the osteochondral interface of OVX mice, which indicated the osteochondral remodeling of the endplate. Here, we speculate that the rapid bone turnover caused by OVX should be responsible for the endplate remodeling and the increased porosity which could reflect the high permeability between vertebrae and the intervertebral disc and which is closely associated with disc degeneration. Several studies could support these etiological hypotheses. Rodriguez et al. [36] found that porosity and permeability of the endplate were increased with age and disc degeneration. Osteoprotegerin (OPG) knockdown mice had an increase in neovascularization and expression of inflammatory cytokines in the intervertebral disc, indicating that osteoporosis can induce inflammation and consequently become the cause of disc degeneration [24]. The modic change of the endplate is closely related to back pain [1, 16]. Endplate damage could also lead to decompression of the nucleus pulposus [6]. Furthermore, severe disc degeneration is more common in patients with endplate modic changes, which suggests that modic changes could result in the occurrence and development of IVDD [45]. Consistently, we found that remodeling of the endplate led to an increased surface porosity and permeability, which could lead to degeneration of the intervertebral disc.

Yuan et al. [42] successfully developed a rat model of IVDD using the injection of alcohol within the endplate to block the blood vessels. IVDD and osteoporosis can also result in endplate cartilage injury [46], supporting that the intervertebral disc interacts with the vertebra. A finite element analysis showed us that both decreased trabecular core density and IVDD have been suggested to play roles in vertebral fractures. IVDD caused a shift of the load from the nucleus pulposus to the anulus fibrosus, resulting in bone adaptation which was presented as a dramatically reduced density of the trabecular core and an increased density in the vertebral walls [47]. Furthermore, the positive correlation between the thickness of the subchondral bone and the proteoglycan content of the adjacent disc have been found in human cadaveric material, particularly in the region of the nucleus pulposus [48]. Bone responds to a greater hydrostatic pressure exerted by discs with higher proteoglycan content than that by discs with less proteoglycan present, which indicates that vertebral osteoporosis with resultant endplate-bone remodeling could affect the flow of solutes to and from the intervertebral disc by failing to maintain the normal hydrostatic pressure of the nucleus pulposus, resulting in the loss of proteoglycans [48]. Therefore, only by guaranteeing a certain appropriate permeability with the proper microstructure of the subchondral bone, being neither too high nor too low, could the endplate maintain the specific hydrostatic pressure and balance the microenvironment of the nucleus pulposus to ensure the stability of the intervertebral disc. The endplate might play the role of a biological semipermeable membrane.

Col II is the most abundant collagen in cartilaginous tissues and is often referred to as the major collagen. Therefore, its content is crucial for proper disc function, particularly in the cartilaginous endplate and nucleus pulposus [49]. Our study showed that OVX could effectively weaken Col II expression, especially in the zone of ossification of the endplate, which could be related to OVX-induced osteochondral remodeling. Furthermore, the levels of OPN and VEGF were markedly elevated in the endplate and annulus fibrosus of OVX mice, consistent with the results of IVDD caused by spinal instability [31]. It is noteworthy that colocalized expression of OPN and VEGF were visible in degenerative discs. The molecular pathological changes in the intervertebral disc indicate that ossification and angiogenesis of the intervertebral disc are not to supply more nutrient for disc repair but to accelerate the intervertebral disc fibrosis and ossification.

Osteoclastogenesis is a prerequisite for osteochondral remodeling, and the osteoclast resorption process is required to degrade subchondral bone and cartilage [50]. Our study found a significant increase in osteoclasts in the subchondral bone and cartilaginous endplate of the OVX group, consistent with the previous findings. Interestingly, alendronate and calcitonin can inhibit osteoclast activity and osteochondral remodeling to extenuate IVDD [26, 27]. Although parathyroid hormone 1–34 has substantial anabolic effects on bone mass and trabecular microarchitecture, nonsignificant effects have not yet been found on disc degeneration [51]. This leads us to suggest that preventing the remodeling of the osteochondral structure caused by the initial osteoclastogenesis after the menopause could be an important aspect in the fight against the occurrence of intervertebral disc degeneration. Furthermore, some reports have confirmed that suppressing osteoclastogenesis and aberrant angiogenesis could blunt IVDD and osteoarthritis [52, 53], which provides further promise for the treatment of IVDD in the future. It may be beneficial to renew the intervertebral disc by balancing bone metabolism and regulating permeability of the endplate.

## Conclusions

In summary, this study uncovered the mystery of the deteriorative effects of OVX on IVDD, clearly showing that OVX can induce and accelerate the progression of disc degeneration. The underlying mechanisms could be related to the destruction of the structural integrity and the function of the vertebra and endplate induced by OVX, both of which are essential structures for maintaining disc function. OVX modulates bone turnover and results in osteochondral interface remodeling which could also influences the expressions of Col II, OSX, OPN, and VEGF to stimulate the disc degeneration. It is conceivable that osteoporosis of vertebrae and endplate remodeling resulting from estrogen depletion may affect the bone marrow microenvironment and endplate permeability which subsequently could alter the metabolism and biomechanics of the intervertebral disc. Simultaneously, the reduction in estrogen caused by OVX could also directly affect the metabolism of the intervertebral disc because of the presence of estrogen receptors in the nucleus pulposus. These changes may be a reasonable explanation for disc degeneration related to osteoporosis. However, our observations are preliminary and need to be further confirmed through clinical trials or additional animal experiments. Based on the above results, intervention for osteoporosis and improvement in the vertebral body and endplate structure may be an effective way to retard IVDD; the mechanisms of IVDD related to osteoporosis need to be further studied.

### Shortcomings and limitations

There are several limitations for this study. First, the findings from the current study are preliminary because of the limited sample sizes and insufficient evidence. The penetration of the endplate lacks intuitive measurement methods and the porosity of the endplate was defined by an indirect index for assessing permeability. Second, our study lacks investigation at different time points and thus we cannot observe progressive changes of vertebrae and discs. Third, although some differentially expressed proteins were found using immunohistochemistry, other methods of detection were not used due to research grant limitations and insufficient mice disc tissue.

## Additional file

Additional file 1: **Figure S1.** Representative mid-coronal images of L4/5 segment and transverse images of L5. The results demonstrate that the trabecular bone structure of L4 and L5 vertebrae are poorer and significantly thinner in OVX mice (blue arrows) suggesting osteoporosis in OVX mice. The caudal endplate shows obviously increased cavities (red arrow) with a more narrowed disc (red asterisk) which may indicate osteochondral remodeling of the endplate and intervertebral disc degeneration in OVX mice. (TIF 592 kb)

## Abbreviations

BMD: Bone mineral density
BV: Bone volume
Col: Collagen
CONN.D: Connectivity density
CT: Computed tomography
H&E: Hematoxylin and eosin
IVDD: Intervertebral disc degeneration
OPN: Osteopontin
OSX: Osterix
OVX: Ovariectomy
PO(op): Open porosity
Po.N(cl): Number of closed pores
Po.V(tot): Total volume of pore space
ROI: Region of interest
SMI: Structural model index
Tb.N: Trabecular number
Tb.Pf: Trabecular pattern factor
Tb.Sp: Trabecular separation
Tb.Th: Trabecular thickness
TRAP: Tartrate-resistant acid phosphatase
TV: Total volume
VEGF: Vascular endothelial growth factor
