# Supplementary figures and images for: Osteoporosis of the vertebra and osteochondral remodeling of the endplate causes intervertebral disc degeneration in ovariectomized mice
Source: Arthritis Res Ther. 2018 Sep 10;20:207. doi: 10.1186/s13075-018-1701-1 (PMC6131954; doi:10.1186/s13075-018-1701-1)

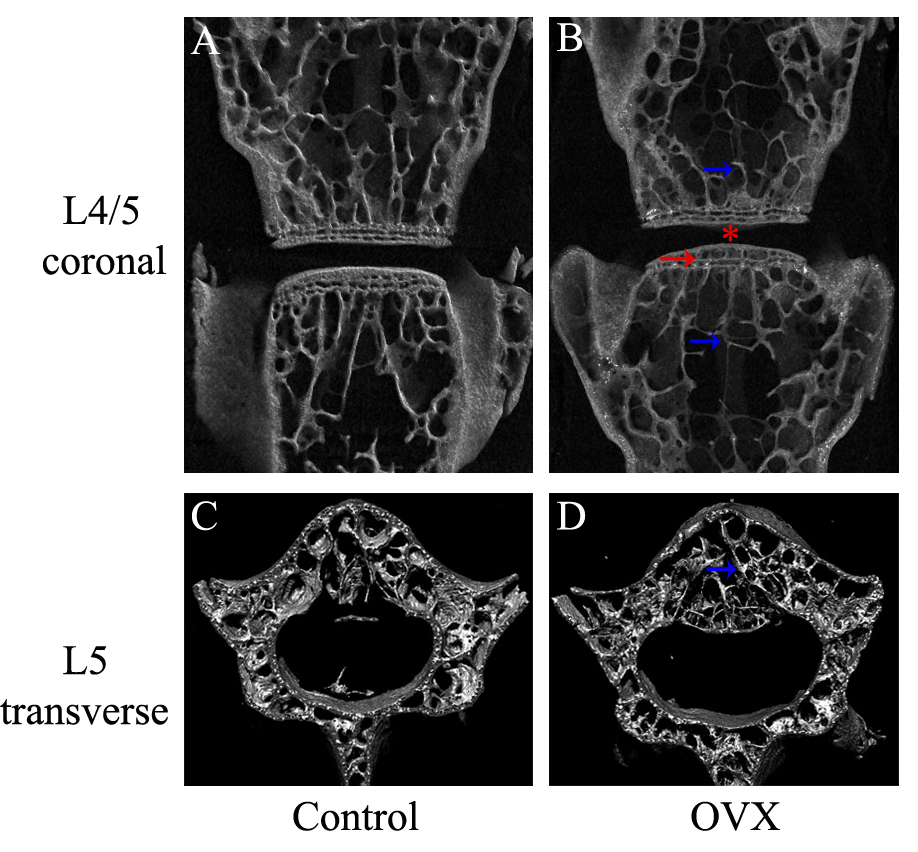

Supplement: Supplementary file 1 — Figure S1. Representative mid-coronal images of L4/5 segment and transverse images of L5. The results demonstrate that the trabecular bone structure of L4 and L5 vertebrae are poorer and significantly thinner in OVX mice (blue arrows) suggesting osteoporosis in OVX mice. The caudal endplate shows obviously increased cavities (red arrow) with a more narrowed disc (red asterisk) which may indicate osteochondral remodeling of the endplate and intervertebral disc degeneration in OVX mice. (TIF 592 kb) [file 13075_2018_1701_MOESM1_ESM.tif]
